# Letter to the Editor of Journal of Otolaryngology regarding “Risk of diabetes in patients with sleep apnea: comparison of surgery versus CPAP in a long-term follow-up study”

**DOI:** 10.1186/s40463-023-00662-5

**Published:** 2023-09-19

**Authors:** Nguyen Truong, Bao Sciscent, F. Jeffrey Lorenz, David Goldrich, Neerav Goyal

**Affiliations:** 1 Penn State College of Medicine, Hershey, PA USA; 2 Department of Otolaryngology–Head and Neck Surgery, Penn State College of Medicine, Hershey, PA USA

**Keywords:** Obstructive sleep apnea, Continuous positive airway pressure (CPAP), Upper airway surgery, Diabetes, Big data

## Abstract

Obstructive sleep apnea (OSA) is associated with multiple chronic comorbidities with treatments including continuous positive airway pressure (CPAP), upper airway surgery (UAS), and hypoglossal nerve stimulation (HNS). Given the complexity of the condition and multiple treatment options, there is an ongoing debate to determine the best management. O’Connor-Reina et al. recently published a paper titled “Risk of diabetes in patients with sleep apnea: comparison of surgery versus CPAP in a long-term follow-up study.” In their study, the authors stated that OSA patients who received surgery had a 50% less chance of developing diabetes compared to patients who only received CPAP treatment. However, we would like to point out some limitations that warrant attention and caution interpretation of the findings by physicians and patients.

## Opening

A recent article was published in the *Journal of Otolaryngology—Head & Neck Surgery* titled “Risk of diabetes in patients with sleep apnea: comparison of surgery versus CPAP in a long-term follow-up study” by O’Connor-Reina et al., highlighting a strong interest in the potential benefits of upper airway surgeries (UAS) in reducing risk of diabetes in patients with obstructive sleep apnea (OSA) [1]. This study utilized a federated de-identified database, TriNetX, that compared the rates of new-onset diabetes and mortality in OSA patients treated with UAS to continuous positive airway pressure (CPAP). The idea behind this study was interesting and contributed to the ongoing discourse on the different treatments of OSA. To further this discussion, we would like to point out some limitations that warrant attention and caution interpretation of the findings by physicians and patients.

## Selection bias

First, it is possible that the findings were confounded by selection bias: i.e. patients in the CPAP cohort had significantly more comorbidities than those who underwent UAS, and were therefore more likely to also develop diabetes. As seen in Table 4 of the study, 33.20% of the CPAP group suffered from obesity compared to 18.00% in the UAS cohort and 49.60% of CPAP patients had hypertension compared to 16.50% of the surgery group. This was a consistent trend for all baseline comorbidities in the study. Even though the study mentioned the significant “differences between age, sex and the presence of comorbidity between both cohorts before matching,” this point may need further emphasis. Risk factors for developing diabetes include obesity, a sedentary lifestyle, and lack of physical activity [2–4]. Furthermore, a sedentary lifestyle and inactivity are also correlated to cardiovascular co-morbidities such as hypertension and dyslipidemia [5]. These are important risk factors that should be considered in propensity score matching while building cohorts. The authors noted that all cohorts were matched for age, sex, and co-morbidities, but the large difference in co-morbidities in the CPAP group indicated that these patients likely had other co-morbidities and behavioral factors that were not considered [6]. While authors matched for many relevant comorbidities, the results may still be biased due to the nature of the retrospective study.

Secondly, we agree with the authors that for a multi-year retrospective study, it is imperative to account for follow-up time in selecting cohorts. O’Connor-Reina et al. ensured that all patients had 5-year follow-up from the date of OSA diagnosis, but we believe that selecting the index event as the diagnosis date of OSA, instead of time of CPAP initiation or surgery date, was another limitation. Index events describe the initial occurrence or presentation of a medical condition. It marks the beginning of a diagnosis or treatment and is different for each patient [7, 8]. Since O’Connor-Reina et al. included data “obtained from up to 20 years ago,” a patient diagnosed with OSA in 2003 may have contributed data to this study during 2003–2008. However, it is possible that they didn’t receive UAS until 2020. Therefore, they would have been incorrectly included in the surgical cohort in this study. As such, we suggest the index date should be set to the date of procedure to compare the efficacy of two treatments [9, 10].

## Coding and cohort queries

The ICD-10-PCS codes for Upper Airway Surgeries listed in Table 1 in the O'Connor-Reina study may nonspecifically represent surgeries for OSA or other diagnoses. Upper airway surgeries have many indications in both children and adults, including chronic bacterial tonsillitis, chronic ear infections, etc. [11–13]. Therefore, it is difficult to be certain that documented upper airway surgeries were intended to treat OSA and not other conditions, as the authors noted in the limitations section. One way to minimize this confounding variable is to set a time relation by linking the diagnosis code of OSA within the same day that patients receive UAS treatments. This way, the specificity of the targeted patient population can be improved.

In reviewing the diagnosis codes utilized to build cohorts, we noticed that the cohorts may not be entirely composed of patients of interest. When capturing patients who were prescribed CPAP, the authors used the codes ICD-10-PCS 5A09357, ICD-10-PCS 5A09457, and ICD-10-PCS 5A09557, noting that two of the codes mandate continuous use for more than 24 h which would not be home CPAP patients (Table 1). In the United States, ICD-10-PCS codes are used only to classify procedures performed in an inpatient setting [14]. Inpatient CPAP treatment is indicated for respiratory distress syndrome and respiratory failure, suggesting a sicker patient population [15–18]. Since O’Connor-Reina et al. utilized the Global Collaborative Network in TriNetX, which captures patients globally, this study likely included hospitalized patients with serious conditions. Even though OSA treatment using CPAP therapy may require hospital titration, solely using ICD-10-PCS codes may exclude the general OSA patients using CPAP at home [19]. This has significant implications on the analysis of comorbidities and mortality. On this note, studies have used CPT codes that more appropriately characterizes regular CPAP use because they require physicians to order a CPAP machine and perform face-to-face patient care like mask fitting, titration pressure, and instruction on how to use the machine [20, 21]. That said, we recognize the challenges in gathering the correct codes for CPAP treatment. Since there is no unifying guideline delineating the coding differences across multiple countries, it is difficult to perfectly identify patients receiving CPAP treatment for OSA in a global retrospective study. We also acknowledge that ICD-10-PCS codes are also primarily used in Europe, while CPT codes are the standard for billing in the United States. As there is no translation for CPT into PCS codes for US data, researchers should be careful in choosing the appropriate codes for their study.

**Table 1:** ICD code for CPAP use. Reproduced from O’Connor-Reina et al. [1]

ICD code	Continuous positive airway pressure use
5A09357	Assistance with respiratory ventilation, less than 24 consecutive hours, continuous positive airway pressure
5A09457	Assistance with respiratory ventilation, 24–96 consecutive hours, continuous positive airway pressure
5A09557	Assistance with respiratory ventilation, greater than 96 consecutive hours, continuous positive airway pressure

## OSA treatment efficacy and TriNetX limitations

One of the advantages of large retrospective databases like TriNetX is its ability to provide a bigger sample size, allowing researchers to study rare diseases on a large scale. However, one of their drawbacks is the dependence on diagnosis and procedural codes. As the the authors pointed out in the limitations section, TriNetX “records for CPAP did not include data for the adherence and acceptance of this therapy.” Studies have shown short term CPAP treatment or poor CPAP adherence does not result in decreased diabetic rates, while CPAP adherence and consistent long term CPAP treatments are associated with decreased risk of diabetes [22–26]. Therefore, without a means to accurately measure patient compliance, the results might be biased. Despite a large difference in risk development of new diabetes diagnosis between CPAP and UAS, readers should take cautions when concluding that surgery has a clinically significant role.

## Updated risk of diabetes methods and results

We developed more comprehensive and balanced cohorts to study the risk of diabetes in patients with OSA. The TriNetX database was queried to identify OSA patients over 18 years of age (ICD-10 G47.30 and G47.33). To ensure surgeries were indicated for OSA, patients with head and neck neoplasms were excluded. Patients with a BMI ≥ 35 kg/m^2^ were recommended to undergo weight loss surgery prior to UAS [27]. Thus, these patients were also excluded from the study. Two cohorts were built based on the ICD and CPT codes to ensure comprehensive coverage of CPAP use or UAS (Table 2). In the CPAP cohort, patients with any head and neck surgeries were excluded. Likewise, any patients in the UAS cohort who received CPAP after the procedure were excluded. Only patients with at least 5 years of follow-up after treatment date were included in the study. To balance for confounding variables, 1:1 propensity score matching was performed on patient demographics (age, sex, race, ethnicity) and co-morbidities (Tables 3 and 4). Baseline characteristic comparison and relative risk analysis were performed. Kaplan–Meier analysis was used to estimate 5-year “survival rate” of not developing diabetes. We would caution the interpretation of this analysis as it does not represent survival, but instead depicts developing the outcome of interest—in this case, developing diabetes [28].

**Table 2:** Updated ICD-10 and CPT codes of CPAP and UAS

Procedures	ICD-10 or CPT code
CPAP	CPT 94660, ICD-10-PCS 5A09357, HCPCS A7034, SNOMED 47545007
Upper airway surgeries	CPT 42145, 42299, 42140, 42281, 21685, 42821, 42836, 42892, 42950, 42826, 21,199, 42870, 41120, 42831; ICD-10-PCS 0CQ3, 0CQM0ZZ, 0CQ33ZZ, 0CQN, 0CQN0ZZ, 0CQ30ZZ, 0CTNXZZ, 0CTN0ZZ, 0C570ZZ, 0CTN, 0CU3, 0CU2, 0NQV3ZZ, 0NSX0ZZ, 0NSX04Z, 0NQT3ZZ, 0NQV0ZZ, 0NQTXZZ, 0CBPXZZ, 0CBP3ZZ, 0CBP0ZZ, 0C573ZZ, 0C57XZZ, 0NQT0ZZ, 0NQVXZZ, 0CQ3XZZ

**Table 3:** Diagnosis characteristics used in propensity score match between CPAP and UAS cohorts

Characteristics	ICD-10 code
Diabetes mellitus	E08-E13
Tobacco use	Z72.0
Overweight, obesity and other hyperalimentation	E65-E68
Other chronic obstructive pulmonary disease	J44
Diseases of the nervous system	G00-G99
Cerebrovascular diseases	I60-I69
Other forms of heart disease	I30-I5A
Ischemic heart diseases	I20-I25
Other and unspecified disorders of the circulatory system	I95-I99
Diseases of veins, lymphatic vessels and lymph nodes, not elsewhere classified	I80-I89
Diseases of arteries, arterioles and capillaries	I70-I79
Pulmonary heart disease and diseases of pulmonary circulation	I26-I28
Chronic rheumatic heart diseases	I05-I09
Acute rheumatic fever	I00-I02
Hypertensive diseases	I10-I16
Diseases of the digestive system	K00-K95
Mental, Behavioral and Neurodevelopmental disorders	F01-F99
Diseases of the genitourinary system	N00-N99
Malignant neoplasms of eye, brain and other parts of central nervous system	C69-C72
Epilepsy and recurrent seizures	G40

**Table 4:** Demographic and clinical characteristics of the study population (n = 65,881)

	Before matching			After matching		
	Continuous positive airway pressure (n = 59,787)	Upper airway surgery (n = 9224)	*P-*Value	Continuous positive airway pressure (n = 6651)	Upper airway surgery (n = 6651)	*P-*Value
Age	61.0 ± 12.7	42.9 ± 13.6	< 0.001	46.3 ± 13.2	46.4 ± 12.4	0.5
Sex						
Male	33,253 (55.8%)	4814 (57.8%)	0.001	3901 (58.7%)	3849 (57.9%)	0.4
Female	26,285 (44.1%)	3508 (42.1%)	0.001	2750 (41.3%)	2801 (42.1%)	0.4
Unknown	31 (0.1%)	10 (0.1%)	0.02	0	10 (0.2%)	0.002
Race						
White	41,427 (69.5%)	5247 (63.0%)	< 0.001	4247 (63.9%)	4246 (63.8%)	1.0
Black or African American	8322 (14.0%)	1362 (16.4%)	< 0.001	1014 (15.2%)	1034 (15.5%)	0.6
Asian	353 (0.6%)	141 (1.7%)	< 0.001	89 (1.3%)	96 (1.4%)	0.6
American Indian or Alaska Native	244 (0.4%)	35 (0.4%)	1.0	33 (0.5%)	32 (0.5%)	0.9
Native Hawaiian or Other Pacific Islander	52 (0.1%)	12 (0.1%)	0.1	10 (0.2%)	10 (0.2%)	1
Unknown	9171 (15.4%)	1526 (18.3%)	< 0.001	1261 (19.0%)	1233 (18.5%)	0.5
Ethnicity						
Not Hispanic or Latino	46,411 (77.9%)	5490 (66.0%)	< 0.001	4560 (68.6%)	4577 (68.8%)	0.8
Hispanic or Latino	2399 (4.0%)	773 (9.3%)	< 0.001	507 (7.6%)	519 (7.8%)	0.7
Unknown	10,759 (18.1%)	2060 (24.8%)	< 0.001	1584 (23.8%)	1555 (23.4%)	0.6
Comorbidities						
Diabetes mellitus	29,077 (48.8%)	1255 (15.1%)	< 0.001	1195 (18.0%)	1227 (18.4%)	0.5
Tobacco use	3045 (5.1%)	190 (2.3%)	< 0.001	160 (2.4%)	177 (2.7%)	0.3
Overweight, obesity and other hyperalimentation	39,864 (66.9%)	3183 (38.2%)	< 0.001	2927 (44.0%)	2885 (43.4%)	0.5
Other chronic obstructive pulmonary disease	16,460 (27.6%)	429 (5.2%)	< 0.001	405 (6.1%)	427 (6.4%)	0.4
Diseases of the nervous system	54,858 (92.1%)	7280 (87.5%)	< 0.001	5782 (86.9%)	5763 (86.6%)	0.6
Cerebrovascular diseases	11,177 (18.8%)	344 (4.1%)	< 0.001	351 (5.3%)	329 (4.9%)	0.4
Other forms of heart disease	36,447 (61.2%)	1636 (19.7%)	< 0.001	1588 (23.9%)	1555 (23.4%)	0.5
Ischemic heart diseases	23,472 (39.4%)	645 (7.7%)	< 0.001	665 (10.0%)	635 (9.5%)	0.4
Other and unspecified disorders of the circulatory system	12,741 (21.4%)	456 (5.5%)	< 0.001	415 (6.2%)	416 (6.3%)	1.0
Diseases of veins, lymphatic vessels and lymph nodes	15,401 (25.9%)	683 (8.2%)	< 0.001	635 (9.5%)	631 (9.5%)	0.9
Diseases of arteries, arterioles and capillaries	15,873 (26.6%)	519 (6.2%)	< 0.001	544 (8.2%)	497 (7.5%)	0.1
Pulmonary heart disease and diseases of pulmonary circulation	13,086 (22.0%)	221 (2.7%)	< 0.001	252 (3.8%)	217 (3.3%)	0.1
Chronic rheumatic heart diseases	6295 (10.6%)	102 (1.2%)	< 0.001	103 (1.5%)	100 (1.5%)	0.8
Acute rheumatic fever	218 (0.4%)	10 (0.1%)	< 0.001	10 (0.2%)	10 (0.2%)	1
Hypertensive diseases	47,055 (79.0%)	3130 (37.6%)	< 0.001	2996 (45.0%)	2984 (44.9%)	0.8
Diseases of the digestive system	45,097 (75.7%)	4965 (59.7%)	< 0.001	4179 (62.8%)	4151 (62.4%)	0.6
Mental, Behavioral and Neurodevelopmental disorders	38,332 (64.3%)	4016 (48.3%)	< 0.001	3461 (52.0%)	3435 (51.6%)	0.7
Diseases of the genitourinary system	41,119 (69.0%)	3717 (44.7%)	< 0.001	3098 (46.6%)	3144 (47.3%)	0.4
Malignant neoplasms of eye, brain and other parts of CNS	235 (0.4%)	21 (0.3%)	0.05	21 (0.3%)	18 (0.3%)	0.6
Epilepsy and recurrent seizures	2661 (4.5%)	258 (3.1%)	< 0.001	239 (3.6%)	225 (3.4%)	0.5

**Fig. 1:**
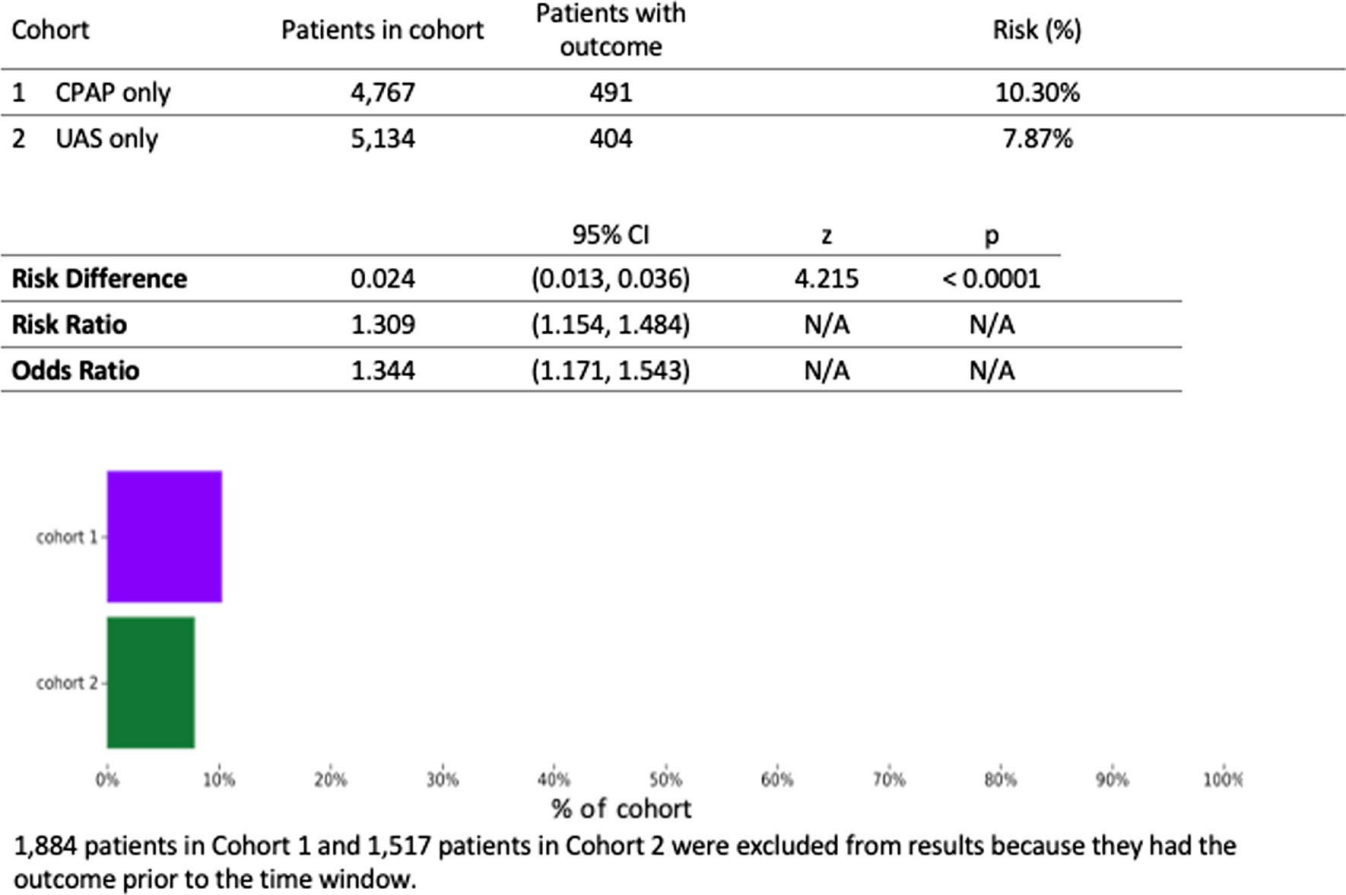
Risk analysis after excluding patients with the outcome (Diabetes) prior to time window

**Fig. 2:**
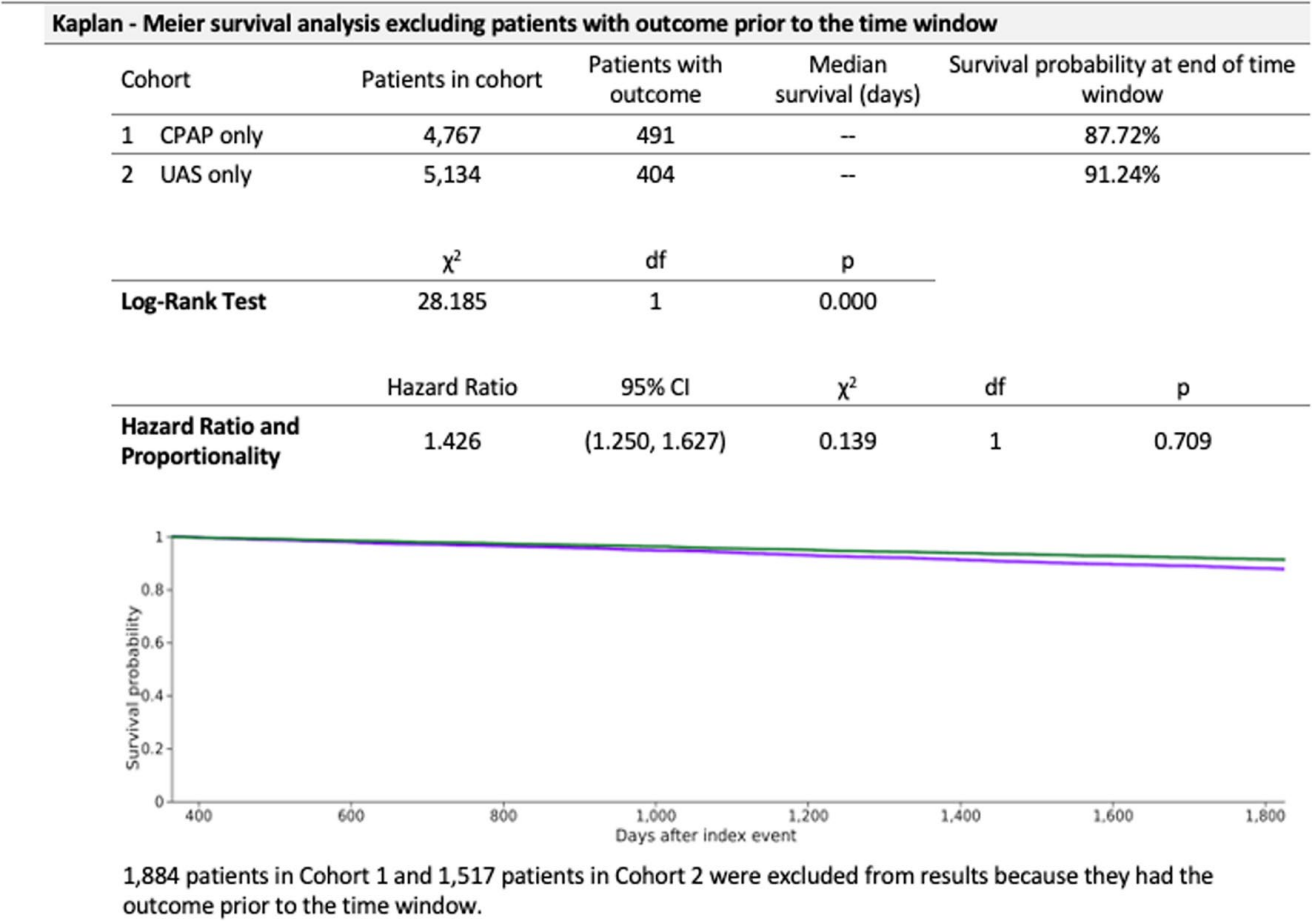
Kaplan Meier plot comparing outcome of diabetes after five years of follow up in both cohorts

Of all patients greater than 18 years of age who had at least 5 years of follow-up after treatment initiation, there were 59,787 and 9224 patients in the CPAP and UAS cohorts, respectively. After 1:1 propensity score matching and excluding patients who did not satisfy inclusion criteria, there were 6651 patients in each cohort. The mean age at index was 46 years of age. Both groups included about 58% male with 64% white, 15% black, and 8% Hispanic. There were 491 (10.3%) patients in the CPAP group and 404 (7.9%) patients in the UAS group with new onset diabetes (Fig. 1). Within the CPAP group, the number of patients with a new diagnosis of diabetes after treatment in the UAS group was significantly lower than the CPAP group (risk ratio RR = 1.31, 95% confidence interval CI = [1.15, 1.48], *p* < 0.0001). Figure 2 shows a 5-year probability of not developing diabetes in CPAP vs UAS cohort (87.7% vs 91.2%, hazard ratio = 1.43, 95% CI = [1.25, 1.63]).

Our results showed that with a comprehensive usage of codes and extensive co-morbidities matching, there is still a statistically significant reduction in the risk of developing new diabetes in the UAS cohort compared to CPAP group, though the absolute risk difference may not be as clinically relevant.

## Conclusion

In conclusion, we feel strongly that there are limitations in the study published by O'Connor-Reina et al. which bias the comparison of UAS versus CPAP. We commend the authors for studying an important topic and we appreciate their consideration of the points we have made here. By facilitating a balanced discussion on this topic, we can advance the understanding and management of OSA.

## Data Availability

The data that support the findings of this study are available from the corresponding author upon reasonable request.

## Abbreviations

OSA: Obstructive sleep apnea
CPAP: Continuous positive airway pressure
UAS: Upper airway surgery
HCOs: Health-care organizations
CI: Confidence interval
BMI: Body mass index
ICD-10-PCS: International classification of diseases procedure coding system
CPT: Current procedural terminology
